# The Strong African American Families Program: Disrupting the Negative Consequences of Racial Discrimination Through Culturally Tailored, Family-Based Prevention

**DOI:** 10.1007/s11121-022-01432-x

**Published:** 2022-09-15

**Authors:** Cady Berkel, Velma McBride Murry, Nalani A. Thomas, Beza Bekele, Marlena L. Debreaux, Catherine Gonzalez, Rachel A. Hanebutt

**Affiliations:** 1 College of Health Solutions, Arizona State University, 425 N. 5th St, Phoenix, AZ 85004 USA; 2 REACH Institute, Arizona State University, Tempe, USA; 3 Departments of Health Policy and Human & Organizational Development, Vanderbilt University, Nashville, USA; 4 College of Health Solutions, Arizona State University, Phoenix, USA; 5 Department of Psychology, Arizona State University, Phoenix, USA; 6 Department of Human & Organizational Development, Vanderbilt University, Nashville, USA

**Keywords:** African Americans, Discrimination, Adolescents, Racial socialization, Evidence-based prevention, Parenting

## Abstract

Racism continues to be a major source of stress for African Americans and can impair psychological functioning. Adolescents experiencing discrimination may engage in self-soothing, but risky behaviors, which leave them at risk for negative life trajectories. Black pride has been identified as a key factor in explaining the heterogeneity in responses to discrimination. Racial socialization, strategies parents use to promote Black pride and protect youth from discrimination, is an important focus of family-based prevention programs serving African American families. This study tests the efficacy of a culturally tailored preventive intervention for rural African American families to disrupt the negative consequences of discrimination on adolescent psychological functioning. Four waves of data from the Strong African American Families (SAAF) efficacy trial (Murry & Brody in Journal of Marital & Family Therapy 30(3):271-283, 2004) with 667 African American families in rural Georgia were used for this study. Structural equation modeling was used to test study hypotheses. Adolescent experiences with discrimination at age 15 predicted concurrent psychological functioning and multiple risk behaviors at age 16, including sexual risk behavior, substance use problems, academic failure, and juvenile justice involvement. Mediation analyses demonstrated that psychological functioning was a significant mediator of these relations. The SAAF program was associated with increases in racial socialization, which in turn fostered gains in adolescent Black pride. Black pride was indirectly associated with reduced risk behavior through adolescent psychological functioning, but Black pride did not moderate the effect of discrimination on psychological functioning. This study confirms that family-based prevention can support African American adolescent mental health in the context of discrimination. However, more emphasis on reducing exposure to discrimination is needed.

## Introduction

Racism continues to be a major source of stress for African Americans, especially in the rural South, where vestiges of slavery and Jim Crow laws produce an unequal distribution of economic, educational, and health resources (Murry et al., 2018). Discriminatory experiences can impair psychological functioning, producing hopelessness, and depression (Gaylord-Harden & Cunningham, 2009; Murry et al., 2021; Smith-Bynum et al., 2014; Yip et al., 2019). African American adolescents regularly confront situations that convey a lack of fairness and justice, which call into question the possibility of future success through traditional pathways (Golden et al., 2018; Kenyatta, 2012). These demoralizing experiences can result in diminished academic self-efficacy and poor achievement outcomes (Brittian & Gray, 2014; Gale & Dorsey, 2020; Griffin et al., 2017; Tang et al., 2016; Thomas et al., 2009; Wang & Huguley, 2012). Discrimination and low expectations for success via academic pathways are risk factors for problem behaviors, which can lead to contact with the juvenile justice system (Gibbons et al., 2020; Tobler et al., 2013; Unnever et al., 2016). Furthermore, discriminatory school policies have directly led to the “school-to-prison pipeline” (Skiba et al., 2014).

To cope with demoralization, adolescents may engage in self-soothing, but risky behaviors, such as sexual behavior or substance use, as a “quick fix” coping strategy (Baumeister & Sher, 1988; Murry et al., 2014). A growing body of evidence links discrimination to HIV risk, including substance use and sexual risk behaviors, for racial and ethnic minority adolescents (Clark et al., 2015; Flores et al., 2010; Gibbons et al., 2012; Kogan et al., 2015; Rosenthal et al., 2014; Tobler et al., 2013). The accumulation of racist experiences over time may explain the “racial crossover effect” where African Americans transition from minimal substance use to rates during adolescence to above the national average in early adulthood (Banks & Zapolski, 2018; French et al., 2002; Trinidad et al., 2004; Watt, 2008). These behaviors can convey risk of acquiring HIV, and indeed, HIV disparities are persistently high for African Americans (Centers for Disease Control & Prevention, 2021).

Despite the negative effects of racism, it is important to recognize heterogeneity in youth outcomes. Racial pride has been identified as a key factor in explaining how some youth thrive despite exposure to discrimination (Caldwell et al., 2004; Scott, 2003; Sellers et al., 2006; Wong et al., 2003). For example, a latent class analysis demonstrated that African American adolescents with high levels of racial pride, self-efficacy, and self-acceptance were more likely to persist in school despite experiences with discrimination (Butler-Barnes et al., 2013). African American parents’ use of racial socialization is a determining factor in nurturing these key elements of resilience (Berkel et al., 2009; Wang & Huguley, 2012). Messages that balance a preparation for discrimination with an emphasis on the strengths of the African American community and ancestral heritage appear to be particularly beneficial. Consequently, racial socialization is thus an important target for family-based prevention programs serving African American families (Anderson et al., 2019; Coard et al., 2004; Murry & Brody, 2004).

The Strong African American Families (SAAF) was developed by Murry and Brody in partnership with African American communities in rural Georgia to prevent disparities for African American youth (Brody et al., 2004; Murry & Brody, 2004). SAAF includes universal parenting skills common to other family-centered evidence-based prevention programs. With guidance from long-standing community partners, SAAF was enriched to include a focus on discrimination and racial socialization (for more details, see Intervention). At the time of its development, there were no other evidence-based programs designed for rural African American families or that included a focus on discrimination and racial socialization (Jones & Neblett, 2016; Murry et al., 2012). In 2001, an efficacy trial was conducted to evaluate SAAF with rural African American families with early adolescents. Previous results of that trial have demonstrated intervention effects on a constellation of adaptive parenting skills, which included universal parenting strategies and racial socialization (Brody et al., 2005, 2006, 2008; Murry et al., 2005, 2007, 2011). These improvements in parenting translated to increases in internal youth assets, including self-regulation, racial identity and pride, and positive body image and sexual self-concept at age 12; and reductions in conduct problems at age 14 and in substance use and early sexual activity at age 14 and 16.

Despite the critical role of discrimination in the heuristic model guiding the SAAF program, no studies to date have explicitly analyzed the impact of discrimination to understand the mechanisms by which the program may disrupt its negative consequences for youth. Nor have studies been undertaken to examine the relative effects of discrimination and the SAAF program on adolescent psychological functioning. Although the literature has clearly demonstrated support for the benefit of racial pride (e.g., Sellers et al., 2006; Yip et al., 2019), the specific processes through which these protective processes buffer youth from succumbing to the negative consequences of discrimination have not been clearly established. SAAF seeks to enhance Black pride during preadolescence, thereby serving as an inoculation against future exposure to discrimination during the adolescent period (Berkel et al., 2009; Greene et al., 2006; Smith-Bynum et al., 2014). It is unknown, however, if the program’s indirect effects through Black pride would be primarily beneficial for adolescents who experience higher levels of discrimination (i.e., selective prevention) or would be equally beneficial for all participants (i.e., universal prevention). This distinction is important for the interpretation of empirical research, but also the design and implementation of preventive interventions.

**Fig. 1:**
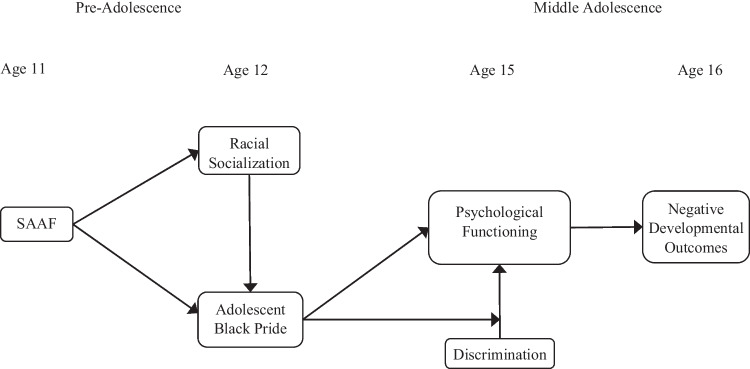
Conceptual model

To address these gaps, the current study tested a set of hypotheses regarding the effectiveness of SAAF in preventing maladaptive coping among rural African American youth in response to discrimination (see Fig. 1). We first hypothesized that exposure to discrimination at age 15 would have indirect effects on a set of key developmental outcomes at age 16 (i.e., substance use problems, sexual risk behavior, academic failure, and justice involvement) through psychological functioning. Next, we examined whether previously established program effects on racial socialization and Black pride would buffer or mitigate those effects. In particular, we examined the direct effect of Black pride at age 12 on psychological functioning at age 15, above and beyond the impact of discrimination. We also examined whether Black pride at age 12 would reduce the negative concurrent impact of discrimination on psychological functioning.

## Methods

### Overview of the Study Design

All study procedures were approved by and conducted with oversight from the IRB at the University of Georgia. All measures and protocols were developed and refined with feedback collected via focus groups with 40 African American members of the local community (Murry & Brody, 2004). The sample for this trial was drawn from nine counties that were selected for participation based on their proximity to the University of Georgia, high representation of African American residents, and rurality (i.e., low population density, declining agrarian economy, and limited access to public transportation and health services). Counties were matched based on size and poverty rates (note that two small counties were combined to create a unit of comparable size to the other seven counties). For each pair, one was randomly assigned to receive the SAAF intervention and the other a literature control, which included educational pamphlets about health and development. Public middle schools in the sampled counties provided African American staff at the Center with rosters of fifth-grade students to be used as recruitment lists. All African American families with 11-year-old children were eligible for participation. No other exclusion criteria were used. Families were randomly selected from the lists for recruitment, resulting in a total sample of 671 families. Participation rates and demographics were similar for intervention and control counties. There was minimal attrition across waves, with 571 participating at Wave 6 (86%); this did not differ significantly by condition (IV: 88%, Control: 82%). So that we could examine how delivery of the program at age 11 would prepare families to cope with discrimination during later adolescence, this study used data from four data collection points, including wave 1 (when children were 11 years old), wave 2 (12 years old), wave 5 (15 years old), and wave 6 (16 years old).

### Participants

There were 2.7 children per family and 54% of the target children were females. Of those families, 30% were 2-parent household and the average parent age was 38 years old. Most (79%) parents completed high school. Families reported working an average of 39.4 h per week and the families’ median household income per month was $1655. In regard to living standards, 46% of families were living below federal poverty standards and 50% were living within 150% of the poverty threshold.

### Intervention

SAAF was the first empirically based program designed specifically for rural African American families. Program content and delivery were guided by a culturally informed heuristic model of risk and protective mechanisms (Barrera & Castro, 2006), which was derived from theory, research, and input from community stakeholders (Brody et al., 2004; Murry et al., 2005). It includes universal parenting strategies found across family-centered preventive interventions (Sandler et al., 2015), as well as culturally specific protective elements (Murry et al., 2007, 2011). Prior to its development, Murry and Brody had conducted over a decade of community engaged research with African American families in rural Georgia (e.g., Brody et al., 1994; Murry & Brody, 1999), which led to an establishment of trust, a nuanced understanding of the local context, and the impetus for the development of a program that would build on cultural strengths to address issues of concern to the local community. The program of research was guided by resilience perspectives (Bogenschneider, 1996; Luthar, 2015), as many adolescents at risk for negative developmental trajectories because of racism and poverty nevertheless are able to overcome these challenges and the competence model of family functioning (Waters & Lawrence, 1993), which points to adaptive parenting as an important contributor to resilience. General and culturally informed theories of adolescent development (e.g., Bandura, 1997; Gibbons & Gerrard, 1997; McAdoo, 1997) informed the programs’ focus on protective factors during the transition to adolescence.

Community stakeholders identified their concerns for their children, including mental health, substance abuse, and sexual risk behavior; these concerns became the distal outcomes of the program (Murry & Brody, 2004). Community input emphasized the need for the program to include a recognition of the negative impact of discrimination on each of these outcomes. It also guided the incorporation of key cultural strengths, including racial socialization, or strategies that parents use to protect children from the negative impact of discrimination, and the Black church, which serves as a trusted organizational feature of family life in the partnering communities.

The program includes concurrent 1 h sessions for parents (led by one facilitator) and children (led by two facilitators), followed by a joint 1 h family session (led by all three facilitators). Parent sessions focus on nurturing parent child-relations, monitoring, consistent and effective discipline, clear expectations regarding substance use, strategies for communicating about sex, and adaptive racial socialization. Child sessions focus on future orientation, prototypes, resistance efficacy, and adaptive behavioral strategies to use when encountering racism. Family sessions focus on communication skills and family cohesion, through games, discussions, and other activities to reinforce the content covered in the individual sessions. Importantly, a focus on overcoming discrimination through racial socialization and Black pride is interwoven throughout the program. For example, each session ends with stating a parent, youth, and family creed (e.g., “African American families care about each other and have fun together. We are making a difference in the world”). The sixth session in particular is explicitly focused on adaptive ways to help children navigate discrimination (Berkel et al., 2013).

### Implementation

Weekly sessions were held at local community centers, churches, schools, or libraries that were familiar and convenient for families. They were led by African American facilitators with previous experience in teaching or working with families and 40 h of training, which included a didactic portion on the theory and background for each session and problem-solving discussions for potentially challenging activities, followed by an opportunity to practice delivering each section. Program sessions were videotaped and coded to assess the extent to which program facilitators adhered to the instructions in the manual. Each instruction in the manual was turned into an item. Most were rated on a dichotomous yes/no scale, although some included a partial completion or a count when appropriate. The score was calculated as the total number of fidelity items completed divided by the total possible for each activity and then the session as a whole. Two sessions per group were randomly selected for coding by a group of coders, whose interrater reliability was 80%, assessed mean program fidelity at 88% (Berkel et al., 2013).

Extensive measures were taken to address potential barriers to program attendance. Community liaisons were employed to serve as bridges between the research team and the communities to facilitate communication and trust (Murry & Brody, 2004). Each session started with a meal catered by a local African American business to encourage participation, provide a buffer for late arrivals, and establish rapport among the facilitators and families in the group. Postcards were sent weekly to explain the upcoming session and follow-up calls were made to families who missed. There were also incentives for regular participation, including monetary incentives and prizes (e.g., family board games). As a result, two-thirds of the families attended at least five of the seven sessions, an attendance rate exceeding that of a similar family-centered program serving 2-parent, middle-class families in the Midwest (Spoth et al., 1998). In most cases, the primary caregiver in attendance was the mother. A total of 29 of the fathers attended at least one session and their mean attendance was 3.5 sessions (SD = 2.1).

### Data Collection

Parents provided consent for themselves and their children. Children provided assent for themselves. Assessments were conducted in the families’ homes by African American interviewers from similar communities who received 27 h of training in conducting computer-based interviews. Parents and children responded to a battery of instruments via laptop computers. Interviews were conducted in separate areas of the house so that other members of the household could not overhear. To maintain confidentiality, while also preventing potential literacy concerns, questions were read via computer using Audio Computer Assisted Self-Interview (ACASI) programming. Interviewers guided participants through the batteries, however, participants entered their own responses to potentially sensitive questions (e.g., substance use, sexual risk behavior) onto a remote keypad so that interviewers could not see them. Interviewers completed comment cards at the end of every session to report on any issues related to adverse events or quality control. Cards were collected and reviewed daily by the project coordinator, who reported any concerns to the PIs. No adverse events occurred during the course of the study. The interviews lasted approximately 2 h for each participant. Families received $100 at the completion of each wave of data collection to reimburse them for their time.

### Measures

#### Condition

Random assignment to the SAAF program was dummy coded, such that intervention families were coded as 1 and control group families were coded as 0.

#### Racial Socialization

Parents reported their racial socialization at waves 1 and 2 (when children were 11 and 12 years old) using the 15-item Racial Socialization Scale (Hughes & Johnson, 2001) which includes Preparation for Bias (6 items), Cultural Socialization (5 items), and Promotion of Mistrust (4 items) subscales. Items were rated using a 3-point Likert scale ranging from 1 (never) to 3 (three to five times). The stem for each question is, “How often in the past month have you…” followed by specific socialization behaviors. Sample item included: “talked with your child about the possibility that some people might treat him/her badly or unfairly because of his/her race” (Preparation for Bias); “taken your child to places or events that reflect racial heritage” (Cultural Socialization); and “told your child not to trust kids from other racial or ethnic groups” (Promotion of the Mistrust, negatively coded). Cronbach’s α was .85 at wave 1 and .89 at wave 2.

#### Black Pride

Adolescent Black pride was measured at waves 1 and 2 (when children were 11 and 12 years old) using an 8-item version of Inventory of Black Identity (Sellers et al., 1997). Items were rated using a 5-point Likert scale with responses ranging from 1 (strongly disagree) to 5 (strongly agree). A sample item from is “I am happy to be Black.” Cronbach’s $\alpha$ were .60 and .67, respectively.

#### Discrimination

Adolescent exposure to discrimination was measured at wave 5 (when children were 15 years old) using 9-items from the Racism and Life Experiences Scale (Murry et al., 2001), which was developed in partnership with community members for this study. Adolescents reported how frequently they experienced racist hassles over the last 6 months with responses ranging from 1 (never) to 4 (frequently). Sample items include “Have you been treated rudely or disrespectfully because of your race?” and “Have you been called a name or harassed because of your race?” Cronbach’s $\alpha$ was .87.

#### Psychological Functioning

Adolescents’ psychological functioning at wave 5 (when children were 15 years old) was modeled as a latent construct using three measures, including hope, depression, and perceived life chances. Hope was measured using a 6-item version of the State Hope Scale (Snyder et al., 1996). Items were rated using an 8-point Likert scale ranging from 1 (definitely false) to 8 (definitely true). Sample items include “At the present time, I am energetically pursuing my goals” and “There are lots of ways around any problem that I am facing now.” Cronbach’s $\alpha$ was .85. Depression was measured using the 26-item Child Depression Inventory (CDI; Helsel & Matson, 1984). Items were rated on a 3-point Likert scale measuring the degree of symptoms. Sample items include “I am sad all the time” and “Nothing is fun at all.” A study examining the factor structure of the CDI among White and Black youth found that it adequately measured depressive symptoms among both populations (Steele et al., 2006). Cronbach’s $\alpha$ was .86. Perceived life chances were measured using the 10-item Measure of Perceived Life Chances (Jessor et al., 1990). Items were rated on a 5-point Likert scale with responses ranging from 1 (very low) to 5 (very high). Each of the items begin with the stem “What are the chances that…?” with sample statements including “you will graduate from high school” and “you will be respected in your community.” Cronbach’s $\alpha$ was .89.

#### Risk Outcomes

Four outcomes were measured at wave 6 (when children were 16 years old) and used to examine effects on distal outcomes that have been linked to adolescent experiences of discrimination and have the potential for effects on negative life-course trajectories. Adolescent substance use problems were measured using a 13-item version of the Minnesota Survey of Substance Use Problems (Harrison et al., 1998). Adolescents reported the frequency with which they had used substances in dangerous situations, failed to meet a responsibility, or experienced legal, social, or interpersonal problems in the last 12 months due to substance use. Responses ranged from 0 (zero) to 6 (11 or more). Cronbach’s $\alpha$ was 0.89. A sexual risk composite index was formed to assess SAAF’s efficacy on youth sexual behavior patterns. The index consisted of three items, one in which youths were asked (a) if they had ever had sex, (b) if they had ever had sex, how frequently did they have sex during the past month, and (c) if they had ever had sex, did they use a condom. Responses were summed, yielding a scale with a possible range of 0–3. Academic failure and justice involvement were measured via two items from the Personal Life Stressor measure, which is a 13-item checklist of potential life experiences, which adolescents rated as to whether they had occurred in the past 12 months. Items used in this study were, “did you fail a class in school” and “have you gotten into trouble with the law.”

### Statistical Analysis

Study hypotheses were tested in Mplus (Muthén & Muthén, 2018). Full Information Maximum Likelihood was used to address missing data (Enders & Bandalos, 2001). ICCs were examined for each variable to determine the potential effect of clustering within group. All were under .05, indicating independence of the data by group (Kreft & de Leeuw, 1998). Multiple fit indices were used to evaluate the adequacy of model fit: either a non-significant *χ*^2^ or a combination of SRMR close to .08, RMSEA close to .08, and/or CFI close to .90, based on simulation studies that revealed using this combination rule resulted in low type I and type II error rates (Hu & Bentler, 1999). We also examined modification indices to determine whether additional paths were indicated by the data. The significance of standardized βs represent tests of study hypotheses. We used bias corrected bootstrap confidence intervals to assess the significance of the standardized indirect effects. Mediation was considered significant if the 95%CI did not cross zero (Fritz & MacKinnon, 2007; MacKinnon et al., 2002; Taylor et al., 2008). To test moderation, predictor variables were centered and an interaction term was created (Aiken & West, 1991).

## Results

### Preliminary Analyses

Means, standard deviations, and correlations for all study variables are presented in Table 1. Racial socialization and Black pride were correlated at age 12, but not age 11. In general, Black pride at age 11 and 12 was associated with positive psychological functioning variables at age 15, whereas discrimination was concurrently associated with poorer psychological functioning. Discrimination was significantly correlated with each of the negative outcomes at age 16, whereas psychological functioning variables were generally associated with less likelihood of these outcomes. To rule out the possibility that psychological functioning caused adolescents to perceive more discrimination, we conducted three cross-lag analyses between discrimination and the psychological functioning indicators at ages 15 and 16. None of the psychological functioning measures predicted increases in discrimination over time. The longitudinal effect from age 15 discrimination to age 16 depression was significant (*β* = .11, **p ≤ .01); longitudinal paths from discrimination to hope and perceived life changes were not significant.

**Table 1:** Correlations and descriptives for study variables

	1	2	3	4	5	6	7	8	9	10	11	12
1. W1 racial socialization	–											
2. W2 racial socialization	.49***	–										
3. W1 Black pride	.04	.03	–									
4. W2 Black pride	.10*	.13***	.26***	–								
5. W5 discrimination	.10*	.07 +	− .06	− .03	–							
6. W5 hope	.01	− .00	.12**	.09*	− .17***	–						
7. W5 depression	− .03	.01	− .06	− .09*	.30***	− .39***	–					
8. W5 perceived life chances	– .06	-.03	.14***	.05	-.13**	.51***	− .32***	–				
9. W6 sexual risk behavior	− .02	− .02	− .02	.06	.15***	− .15**	.10*	− .09 +	–			
10. W6 substance use problems	.09	.08	− .01	− .04	.29***	− .17**	.20**	− .19**	.10	–		
11. W6 academic failure	.04	.02	− .02	− .07	.11**	− .19***	.21***	− .11*	.08 +	.07	–	
12. W6 justice involvement	.06	.07	− .04	− .06	.16***	− .16***	.08 +	− .19***	.20***	.33***	.11**	–
Means	24.00	24.58	12.14	12.50	3.61	25.63	6.80	46.11	8.79	1.52	1.36	1.10
SD	5.89	5.74	2.83	2.46	3.53	3.03	5.98	4.90	16.32	5.12	0.63	0.31

^***^p ≤ .001; ^**^p ≤ .01; ^*^p ≤ .05; + *p* ≤ .10

### Test of the Hypothesized Model

**Fig. 2:**
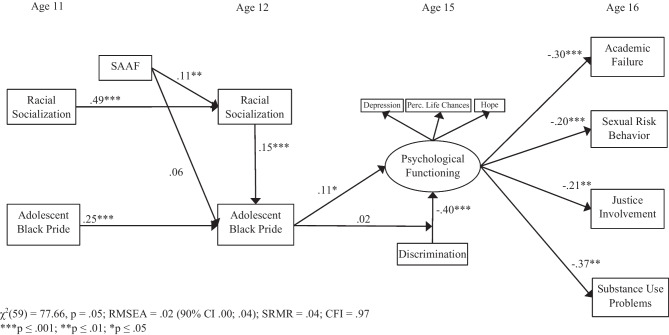
Test of the theoretical model

Results demonstrated good overall model fit [*χ*^2^(59) = 87.50, *p* ≤ .01; RMSEA = .03 (90% CI .02; .04); SRMR = .04; CFI = .97]. Loadings of the hope, perceived life chances, and depression variables for the psychological functioning latent construct were all above .45, *p* ≤ .001. Standardized βs represented tests of study hypotheses (see Fig. 2). Adolescent experiences with discrimination at age 15 were associated with concurrent psychological functioning and predicted negative outcomes at age 16. Mediation analyses demonstrated significant indirect effects of discrimination on sexual behavior (.081; 95% CI = .022; .155), substance use problems (.146; 95% CI = .023; .310), academic failure (.121; 95% CI = .062; .198), and justice involvement (.084; 95% CI = .016; .175).

Random assignment to the SAAF program was not directly associated with increases in adolescent Black pride. However, assignment to SAAF did result in an increase in parents’ use of racial socialization, which in turn, predicted increases in Black pride from age 11 to age 12. This indirect effect of the program on Black pride was significant (.016; 95%CI = .005; .033). Black pride, in turn, predicted psychological functioning over a 3-year period. The indirect effects of age 12 Black pride on age 16 outcomes were also significant: sexual behavior (− .021; 95% CI = − .047; − .003), substance use problems (− .039; 95% CI = − .101; − .006), academic failure (− .032; 95% CI = − .076; − .005), and justice involvement (− .022; 95% CI = − .060; − .003). The Black pride by discrimination interaction term was not significantly associated with psychological functioning, indicating that adolescents benefitted from Black pride, regardless of their experiences with discrimination. It also suggests that discrimination was detrimental regardless of the level of Black pride.

## Discussion

Racism is a complex problem embedded in entrenched policies, practices, and beliefs that perpetuate widespread oppression and unfair treatment of minoritized people. That racism permeates structures and system suggests that dismantling it at the societal level has, thus far, proven to be relatively intractable. During the slow and sporadic progress in reducing the perpetration of discrimination, African American parents are left to prepare their children as best they can by using strategies to buffer them from the negative consequences of racism. Racial socialization reflects an approach developed by African American parents to support children’s development in an unjust social context. Over the past two decades, there has been a growing recognition of the importance of racial socialization as a core component in prevention programs for African Americans and members of other minoritized populations (Anderson & Stevenson, 2019; Anderson et al., 2019; Caldwell et al., 2010; Coard et al., 2004; Yasui & Dishion, 2007). This study was the first to empirically test the ways in which a culturally tailored preventive intervention for rural African American families might support adolescents in overcoming the negative consequences of discrimination.

Results demonstrated significant indirect program effects on psychological functioning and risk outcomes, but a non-significant interaction between Black pride and discrimination. This suggests that Black pride is universally beneficial regardless of exposure to discrimination and should therefore be included in all preventive interventions for African American adolescents. It is important to note, however, that program effects on Black pride were not enough to fully alleviate the negative impact of discrimination over time. Indeed, the relative size of the associations suggests more work is needed, perhaps in the form of booster sessions closer to the time that adolescents engage in risk behaviors driven by discrimination. The most important takeaway, however, is the urgent need to reduce exposure to the deleterious experiences of discrimination. Thus, greater consideration is needed with respect to the ways in which upstream systemic policies, practices, and programs create and perpetuate racism and oppression, creating toxic waters that filter downstream with cascading effects on the everyday life experiences of African American families.

Findings demonstrated that increased exposure to discrimination was negatively correlated with all study outcomes, including diminished hope and perceived life opportunities, increased depression, academic failure, sexual risk behavior, justice involvement, and substance use problems. This is an important finding in that it clearly demonstrates the wide-ranging impact of discrimination, elevating youth risk for both internalizing, and externalizing behavioral problems. Furthermore, this finding contributes to the body of evidence suggesting that the multiple health disparities experienced by African American adults are likely driven by this underlying cause, in this instance, exposure to racial discrimination (Williams & Mohammed, 2009). After decades of urgent calls to action from a select group of research scholars (Black et al., 1988; Braveman & Gottlieb, 2014; Williams & Mohammed, 2009), racial discrimination is finally gaining recognition as a social determinant of health and an adverse childhood experience (Bucci et al., 2015; Slopen & Heard-Garris, 2021). Moreover, the fact that psychological functioning fully mediated associations between discrimination and multiple risk outcomes reinforces the theory that the behaviors are driven by maladaptive coping or attempts to self-soothe societally inflicted psychological wounds (Murry et al., 2014).

A final note is that this study replicates previous findings with respect to the relative influence of parent, adolescent, and family components of the program. We found that the SAAF program’s effects on Black pride were mediated through racial socialization and that the direct effects were not significant. Attempts to scale-up evidence-based prevention programs frequently include a reduction in dosage to improve feasibility for community organizations. In the case of SAAF, the limited direct effects on adolescent outcomes suggest that the adolescent component may be less effective. This has implications for scale-up in the sense that parent-only programs may be more feasible for community organizations to deliver than programs incorporating both parent and child components. Parent engagement may also be more feasible. However, the SAAF program was tested as a complete package, and the impact of the family session, in particular, cannot be ruled out. Eliminating adolescent involvement without more rigorous evidence may result in the weakening of program effects. Dismantling designs should be used to empirically inform program adaptation (e.g., Sandler et al., 2016) to avoid potential voltage drop.

### Limitations

Although this study has many strengths, there are also limitations to note when interpreting the findings. First, while a strength of the study is the fact that the measure of discrimination was developed with community stakeholders based on common, personal experiences of discrimination (Murry et al., 2001), it paints a broad stroke. The field has moved towards more specific measures that reflect a given relationship or context (e.g., discrimination from teachers), which enable a more nuanced perspective linking discriminatory experiences with specific life outcomes (Alliman-Brissett & Turner, 2010; Benner & Graham, 2013; Gale & Dorsey, 2020; Hughes et al., 2016). Nonetheless, this broad measure was highly correlated with all study outcomes. Academic failure and justice involvement were both measured by single items from a larger measure of stressful life events. While this is a limitation, it also speaks to the potential for the use of brief measures that can be used as feasible outcomes for evaluating programs that are scaled up in community settings (Glasgow & Riley, 2013). Finally, some of the study pathways were concurrent, rather than longitudinal. The decision about which timepoints to include was driven by the higher priority of determining how program effects on Black pride during preadolescence would lay a foundation to deter the effect of discrimination during middle adolescence when problem behaviors emerge. Consistent with prior work (Berkel et al., 2010), cross-lag analyses disconfirmed the alterative hypothesis that psychological functioning drives perceptions of discrimination. However, more longitudinal efficacy or effectiveness studies that include culturally specific risk and protective mechanisms are needed to further disentangle the timing of effects.

## Conclusion

African American adolescents’ experiences with discrimination diminish psychological functioning, setting up negative life course trajectories that lead to health and social disparities across a wide array of outcomes. This study confirms that family-based prevention can enhance parents’ tools for protecting adolescents from the consequences of discrimination and that racial socialization and Black pride are universally beneficial, regardless of exposure to discrimination. Nonetheless, effects of discrimination were not fully mitigated. Additional work is needed to prevent exposure to discrimination, and the associated disparities, for African American adolescents.
