# Correction: The Strong African American Families Program: Disrupting the Negative Consequences of Racial Discrimination Through Culturally Tailored, Family-Based Prevention

**DOI:** 10.1007/s11121-024-01699-2

**Published:** 2024-06-14

**Authors:** Cady Berkel, Velma McBride Murry, Nalani A. Thomas, Beza Bekele, Marlena L. Debreaux, Catherine Gonzalez, Rachel A. Hanebutt

**Affiliations:** 1 College of Health Solutions, Arizona State University, 425 N. 5th St, Phoenix, AZ 85004 USA; 2 REACH Institute, Arizona State University, Tempe, USA; 3 Departments of Health Policy and Human & Organizational Development, Vanderbilt University, Nashville, USA; 4 College of Health Solutions, Arizona State University, Phoenix, USA; 5 Department of Psychology, Arizona State University, Phoenix, USA; 6 Department of Human & Organizational Development, Vanderbilt University, Nashville, USA

**Correction to: Prevention Science (2022) 25:44–55** 10.1007/s11121-022-01432-x

The article “The Strong African American Families Program: Disrupting the Negative Consequences of Racial Discrimination Through Culturally Tailored, Family-Based Prevention”, written by Berkel, C., Murry, V.M., Thomas, N.A., Bekele, B., Debreaux, M.L., Gonzalez, C., and Hanebutt, R.A., was originally published Online First without Open Access. After publication in volume 25, issue 1, page 44–55 the author decided to opt for Open Choice and to make the article an Open Access publication. Therefore, the copyright of the article has been changed to © The Author(s) 2022 and the article is forthwith distributed under the terms of the Creative Commons Attribution 4.0 International License, which permits use, sharing, adaptation, distribution and reproduction in any medium or format, as long as you give appropriate credit to the original author(s) and the source, provide a link to the Creative Commons licence, and indicate if changes were made. The images or other third party material in this article are included in the article’s Creative Commons licence, unless indicated otherwise in a credit line to the material. If material is not included in the article’s Creative Commons licence and your intended use is not permitted by statutory regulation or exceeds the permitted use, you will need to obtain permission directly from the copyright holder. To view a copy of this licence, visit http://creativecommons.org/licenses/by/4.0/.

The original article has been corrected.

